# Unconventional fractional quantum Hall effect in monolayer and bilayer graphene

**DOI:** 10.1080/14686996.2016.1145531

**Published:** 2016-04-11

**Authors:** Janusz Jacak, Lucjan Jacak

**Affiliations:** ^a^ Institute of Physics, Wrocław University of Technology, Wyb. Wyspiańskiego 27, 50-370, Wrocław, Poland.

**Keywords:** Monolayer graphene, bilayer graphene, FQHE, hierarchy of filling fractions, 40 Optical, magnetic and electronic devicematerials, 104 Carbon and related materials, 105 Low-Dimension(1D/2D) materials

## Abstract

The commensurability condition is applied to determine the hierarchy of fractional fillings of Landau levels in monolayer and in bilayer graphene. The filling rates for fractional quantum Hall effect (FQHE) in graphene are found in the first three Landau levels in one-to-one agreement with the experimental data. The presence of even denominator filling fractions in the hierarchy for FQHE in bilayer graphene is explained. Experimentally observed hierarchy of FQHE in the first and second Landau levels in monolayer graphene and in the zeroth Landau level in bilayer graphene is beyond the conventional composite fermion interpretation but fits to the presented nonlocal topology commensurability condition.

## Graphical Abstract

**Figure:**
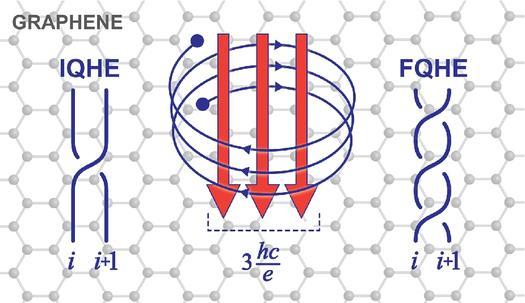

## 1. Introduction

Recent progress in Hall experiments with graphene has revealed many new features in longitudinal and transversal resistivity in Hall configurations characteristic for fractional quantum Hall effect (FQHE), both in suspended graphene scrapings [1–3], and in graphene samples on crystalline substrate of boron nitride [4,5]. The different structure of Landau levels (LLs) in graphene in comparison to the conventional semiconductor two-dimensional electron gas is the source of a distinct scheme for integer quantum Hall effect (IQHE) in graphene, referred to as the ‘relativistic’ version [6]. The Berry phase induced shift for chiral carriers in graphene together with the four-fold spin-valley degeneracy of LLs result in $\nu =4\left(n+\frac{1}{2}\right)$ series for fillings at which IQHE plateaus occur in subsequent centers of LLs for the case of monolayer graphene. When *SU*(4) spin-valley symmetry is broken by a larger magnetic field, the new features for IQHE appear, corresponding to removal of LL subbands degeneracy [7]. In the bilayer graphene an extra degeneracy of n = 0 and n = 1 LLs shifts the IQHE plateau positions to the ends of consecutive LLs, because of eight-fold degeneracy of the lowest LL (LLL) in this case. The higher LLs are still fourfold-degenerate in bilayer graphene. Simultaneously, more and more features at fractional fillings of LLs are observed related to FQHE, revealing also its specific character mostly connected with the different structure of subbands of LLs and particle-hole symmetry due to meeting of the conductivity and valence bands in Dirac points. The new filling fractions for FQHE are observed in the six first subbands of LLs with n=0 and n=1 in monolayer graphene [2–5]. Especially interesting is an observation of unusual even denominator fillings for FQHE in bilayer graphene, including the most pronounced feature at $\nu =-\frac{1}{2}$ [1].

In the present paper we analyze the hierarchy for fractional fillings linked to strongly correlated multiparticle states in graphene using the topological commensurability approach developed earlier for the ordinary 2DEG Hall systems [8–10]. In this way we explain the structure of fractional fillings of LL subbands and demonstrate its evolution with growing number of LL and its subband. This approach, described in [11,12], gives the hierarchy of FQHE in agreement with the available experimental data for monolayer and bilayer graphene. In particular the explanation of the even denominator filling ratios for bilayer graphene, with the FQHE state at $\nu =-\frac{1}{2}$, has been achieved by application of the commensurability topological method to the double layer Hall system.

## 2. Commensurability condition

The concept of the commensurability of cyclotron trajectories with interparticle spacing in a 2D charged system at strong magnetic field is born in relation to the braid group approach to multiparticle systems in the presence of a magnetic field, and employs the interaction of electrons, essential for formation of any correlated state, including states corresponding to FQHE. The collective behavior of a quantum multiparticle state must be assigned by the statistics phase shift acquired by the multiparticle wave function if one considers position exchanges of particle pairs [8,13]. The exchange ofparticles is understood as exchange of the position-arguments of the multiparticle wave function. This quantum feature is associated with the one dimensional unitary representation (1DUR) of the full braid group related to the system. The full braid group is the first homotopy group of the multiparticle configuration space, and topologically classifies all possible particle exchanges. This group is denoted as $\pi_{1}\left(\Phi \right)=\pi_{1}\left(\left(M^{N}-\Delta \right)/S_{N}\right)$, where $M^{N}$ is the *N*-fold normal product of the manifold $M=R^{2}$ for the plane, Δ is the diagonal point set in this product (when coordinates of two or more particles coincide, subtracted in order to assure conservation of the number of particles), $S_{N}$ is the permutation group of *N* elements [14]. The quotient structure of the configuration space $\Phi =\left(M^{N}-\Delta \right)/S_{N}$ is related with the indistinguishability of quantum identical particles – the property necessary for the quantum reason, when renumeration of particles has no effect. The full braid group $\pi_{1}\left(\Phi \right)$ is a topological object collecting all classes of nonhomotopic trajectory loops in the configuration space Φ, where points which differ only by enumeration of particles are unified. Any details of the dynamics of an *N* particle system resulting in special shapes of trajectories are not important here, unless they cause the topological nonequivalence. Only topology of trajectories on *M* decides whether one trajectory loop can be continuously transformed into another one without a cut or not. If a smooth continuous transformation is impossible between two trajectories, they are topologically nonequivalent and fall into distinct classes of the full braid group. Thus, the full braid group does not reflect the dynamics details, but rather identifies the topology restrictions imposed on the multiparticle system, which are associated to some global features of the system including the manifold type and only those aspects of interaction that might cause topological nonequivalence of trajectories. The Coulomb repulsion of electrons on the plane is a central prerequisite for the braid group definition at magnetic field presence, because the scale of interparticle separation defined by the Coulomb repulsion must interfere with the planar cyclotron orbits, discriminating in this way possible correlation types. As the loops from $\pi_{1}\left(\Phi \right)$ describe exchanges of particles the full braid group contains information on quantum statistics of particles, although classical particles do not have any such statistics. It was proved [13] that the 1DURs of the full braid group serve as the determinant of the quantum statistics of particles. Therefore, if particle classical positions given by arguments of the multiparticle wave function $\Psi (x_{1},...,x_{N})$ ($x_{i}$ is the coordinate of *i*-th particle on the manifold *M*, i.e., the classical position of the *i*-th particle on *M*) are changing along a selected loop from the $\pi_{1}\left(\Phi \right)$, then this wave function acquires the phase shift $e^{i\alpha }$ defined by the 1DUR of this particular braid [15]. In this way the statistics of quantum particles can be identified. For the same classical particles the quantum different particles can be defined as assigned by distinct 1DURs of the related full braid group. For three dimensional space, $M=R^{3}$, the full braid group is simply the permutation group $S_{N}$, with only two different 1DURs [14]:

(1) $$\begin{array}{c} \sigma_{i}\to \left\{\begin{array}{c} e^{i0}=1,\\ e^{i\pi }=-1, \end{array}\right. \end{array}$$

where $\sigma_{i}$ is the generator of $S_{N}$, i.e. the elementary braid describing exchange of *i*-th and i+1-th particles, whereas other particles are left in their positions. These two 1DURs correspond to bosons and fermions, respectively. For two-dimensional manifolds *M* the braid groups differ considerably from the permutation group. For $M=R^{2}$, $\pi_{1}\left(\Phi \right)$ is an infinite group with the 1DURs [14]:

(2) $$\begin{array}{c} \sigma_{i}\to e^{i\alpha },\;\alpha \in \left[0,2\pi \right), \end{array}$$

corresponding to so-called anyons (including also bosons for α=0 and fermions for α=π). In this way the unique topology of 2D space causes the substantial change in quantum statistics available for particles in comparison to the 3D space.

**Figure 1.**
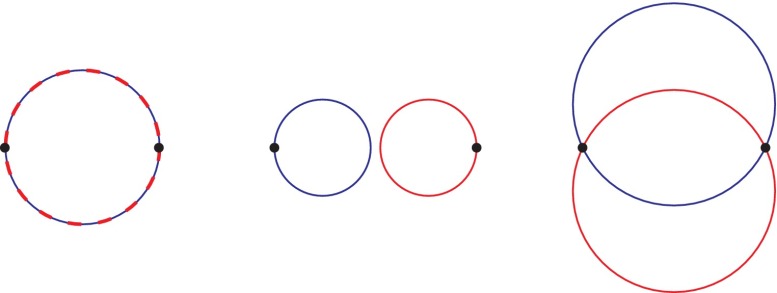
Schematic demonstration that commensurability (left) of cyclotron orbit with interparticle separation satisfies topology requirements for braid interchanges in uniformly and equidistantly (due to interaction) distributed 2D particles; for smaller cyclotron radii particles cannot be matched (center), for larger ones the interparticle distance cannot be conserved (right).

There are, however, also other important consequences of 2D topology, which were not accounted for by the anyon concept. The special feature of planar multiparticle systems manifests itself in the presence of the perpendicular magnetic field strong enough that the classical cyclotron orbit is shorter in comparison to the interparticle separation on the plane. Because the classical trajectories of charged particles are defined by cyclotron orbits at the presence of a magnetic field, thus exchanges of neighboring particles on the plane are possible only if the size of the cyclotron orbit fits to the separation between particles, as is illustrated in Figure 1. Let us emphasize that the separation between particles on the plane is fixed by the Coulomb interaction between particles preventing one particle approaching another in the case of uniformly distributed *N* particles on the plane. This highlights the fundamental role of the electron interaction in formation of any correlated states, including those which can be characterized in topological commensurability terms. The commensurability between the cyclotron radius and the interparticle spacing is required for the definition of the generators $\sigma_{i}$ of the full braid group, where $\sigma_{i}$ describes exchange of *i*-th and i+1-th particles. If the cyclotron orbit is incommensurate with the separation between particles on the plane, then the classical trajectories describing $\sigma_{i}$ elements of the full braid group are impossible and this braid group cannot be implemented. This means that the quantum statistics cannot be defined in this case and any correlated multiparticle quantum state cannot be organized. In this way the possibility for the definition of particle exchanges (i.e. the definition of the braid group andthe quantum statistics) decides whether the quantum correlated collective state can be organized.

The situation when cyclotron orbits are shorter than interparticle separation happens at fractional filling of the LLL. For magnetic fields larger than this one which corresponds to the completely filled LLL, the classical cyclotron orbits are too short to match neighboring particles and the definition of the generators of the full braid group is precluded. Nevertheless, at some ‘magic’ fractional fillings of the LLL the correlated states are experimentally observed and referred to as FQHE [16–18]. This means that possibility of particle exchanges is recovered somehow. In the framework of the composite fermion (CF) approach [19] the enlargement of cyclotron orbit size is achieved by screening of the external magnetic field by auxiliary field flux quanta attached to particles. This artificial model allows for identification of the main line of fractional fillings for FQHE by mapping of the fractional state onto integer quantum Hall states in the resultant magnetic field reduced by the average field of fluxes pinned to CFs. The CF model does not explain neither the origin of auxiliary field fluxes nor the mechanism of creation of composite particles and the construction is rather formal.

**Figure 2.**
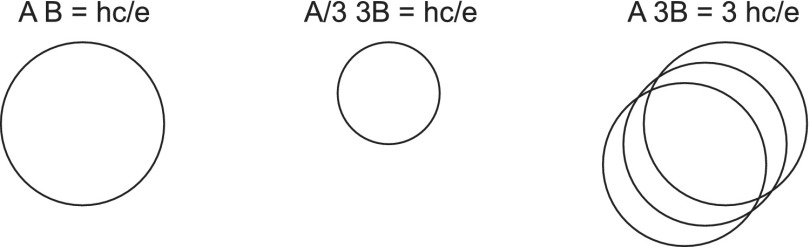
Schematic illustration of cyclotron orbit enhancement in 2D due to multi-loop trajectory structure (third dimension added for visual clarity).

The manifestation of FQHE can be explained, however, in the braid group terms [9]. Though the generators $\sigma_{i}$ of the full braid group cannot be defined when the cyclotron orbits are shorter than interparticle separation, there exist other braids which in 2D fit to interparticle separations. This exceptional property of planar multiparticle systems possess multilooped cyclotron braids, i.e., $\sigma_{i}^{q}$, where *q* is an odd integer, and for e.g. q=3, $\sigma_{i}^{3}$ describes the braid for exchange of the *i*-th and i+1-th particles with one additional loop. Exclusively in 2D the multilooped cyclotron orbits have larger size which can fit to interparticle separation at the ‘magic’ fractional fillings of the LLL, the same ones at which FQHE is observed. The reason for the enhancement of the size of planar multilooped orbits is linked with constant surface spanned by 2D orbits despite its multilooped character (in opposition to the 3D case when each additional loop adds also a surface portion spanned by this loop). When the total external field flux is passing through the 2D multilooped orbit it must be shared among all loops. Thus the flux portion per each loop diminishes, resulting in their size growth. This is illustrated in Figure 2.

In Figure 2 (left) the scheme of the cyclotron orbit at magnetic field *B* is pictorially shown as accommodated to the quantum of the magnetic field flux, i.e. $BA=\frac{hc}{e}$. This serves as the definition of the cyclotron orbit size *A* because this surface *A* fits to the interparticle separation in the case of the completely filled LLL, $\frac{S}{N}$, *S* is the sample area, *N* is the number of particles. If only single-looped orbits are considered, then at, for instance, three times larger field, 3*B*, the cyclotron orbit accommodated again to the flux quantum is too short in comparison to the interparticle separation $\frac{S}{N}$ (which fits to the *B* field orbits but not to 3*B* orbits). This is illustrated in the central panel of Figure 2. Nevertheless, if tree-loop orbits are considered, then in the flat geometry of 2D space, the external flux 3*BA* must be shared among three loops with the same surface *A* (i.e. *BA* for each loop). Thus, each loop accommodated to the flux quantum $\frac{hc}{e}$ has the orbit with the surface *A* and gives the contribution *BA* to the flux, resulting in the total flux 3*BA* per particle, as needed – this is schematically illustrated in Figure 2 (right). The size of *A* in the right panel is equal to *A* in the left panel, which means that the three-loop orbits fit to the interparticle separation.

**Figure 3.**
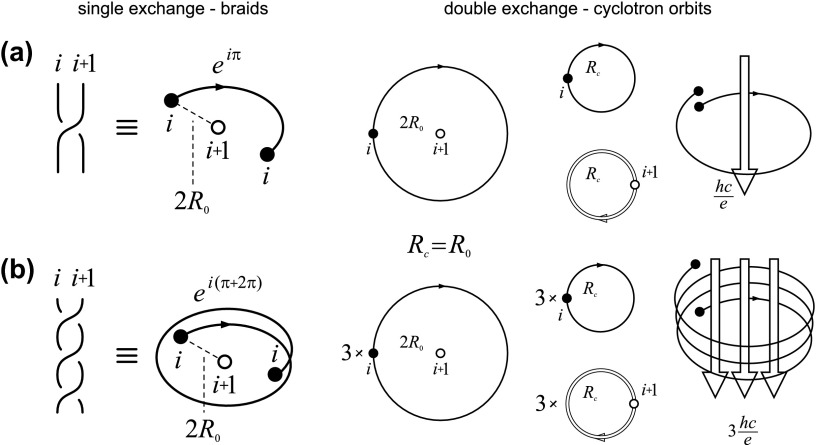
The braid generator $\sigma_{i}$ corresponds to the single exchange of particles (left), the cyclotron orbit (relative) corresponds to the double exchange (right), for (a) ν=1 when single-looped cyclotron trajectory reaches neighboring particles, $R_{c}=R_{o}$; and (b) braid generator $\sigma_{i}^{3}$ for $\nu =\frac{1}{3}$ with additional loop needed for $R_{c}=R_{o}$ ($R_{c}$ is cyclotron radius, $2R_{o}$ is particle separation, i.e. $\pi R_{c}^{2}=\frac{hc}{eB}$, $\pi R_{o}^{2}=\frac{S}{N}$).

As the braid group generator must be defined by the half of the cyclotron orbit (cf. Figure 3) thus the braid with one additional loop corresponds to the cyclotron orbits with three loops – such a generator has the form $b_{i}^{\left(3\right)}={\left(\sigma_{i}\right)}^{3}$. The group generated by $b_{i}^{\left(3\right)},$ i=1,⋯,N (new elementary braid exchanges) is the subgroup of the original full braid group. This subgroup is called the cyclotron braid subgroup and its 1DURs define statistics of 2D charged particles at strong magnetic field corresponding to fractional filling $\nu =\frac{1}{3}$ of the LLL, giving rise to the explanation of the Laughlin statistics for FQHE at this fractional filling (the exponent p=3 in the related Jastrow polynomial in Laughlin function [17]). The generalization to more loops attached to the braid generator one by one, results in double increase of loop number in multilooped cyclotron orbits and thereby in fractions $\nu =\frac{1}{p}$, p-oddinteger. This approach successfully reproduces the hierarchy of the experimentally observed filling fractions corresponding to FQHE in the LLL and in the higher LLs for 2DEG in conventional semiconductors [10].

If $\frac{S}{N}<A$, i.e. when cyclotron orbits are larger than interparticle separation (as in the right panel in Figure 1), some special commensurability opportunity important for braid group definition also occurs. For the filling fractions when $x\frac{S}{N}=A$; x-integer, the cyclotron orbits fit to every *x*-th particle separation ($x\frac{S}{N}=\frac{S}{N/x}$), which also allows for the definition of the generators $\sigma_{i}$ in the form of ordinary single-looped braids, similarly as it happened for the completely filled higher LLs. The related statistics is the same as for IQHE though at some fractional fillings of higher LLs, which is demonstrated in [10] in good correspondence with the experimental observations available up to the third LL for 2DEG in conventional semiconductor Hall systems [20–22]. This situation of too large cyclotron orbits in comparison to particle separation may happen only in such LL subbands where the condition $x\frac{S}{N}=A$ (x-integer) can be fulfilled. It may happen only for n≥1 where *n* is the number enumerating LLs, because the cyclotron orbit size in *n*-th LL grows by factor 2n+1, i.e., $A=\frac{(2n+1)hc}{eB}$ for particles with kinetic energy $\left(2n+1\right)\frac{eB}{2mc}$ in *n*th LL. Simultaneously, for n≥1 too short cyclotron orbits may be encountered only close to subbands edges, for sufficiently small planar density of particles in the subband, because in the higher LLs the cyclotron single-looped orbits are larger as accommodated to higher kinetic energy. This pushes FQHE(multiloop) features in higher LLs toward the edges of subbands in LLs with n≥1, whereas in the central regions of these subbands the new fractional features occur related with the single-looped cyclotron orbits and thus with IQHE-type of correlations but at fractional filling rates. These correlated states are referred to as FQHE(single-loop). The quantization of the transverse resistance $R_{xy}$ related to these fractional filling rates ν of higher LLs is as for ordinary FQHE, $\frac{h}{e^{2}\nu }$, but the correlations of Laughlin type are with the exponent p=1 in the Jastrow polynomial, displaying single-looped braid exchanges like in IQHE. The number of these new fractional filling features grows as 2*n* with the LL number.

## 3. Hierarchy for FQHE in graphene

In graphene one deals with the relativistic version of LLs [6,23]. This is due to the specific band structure in this material being a gapless semiconductor with points *K* and $K^{\prime }$ on the border of the hexagonal Brillouin zone where the valence and conduction bands meet [6]. Thus the low energy particle-hole excitations can be described by the effective Dirac Hamiltonian corresponding to cone-shape of both bands close to the meeting points. The quantization due to presence of the magnetic field has the form of degenerated LLs, though with spectrum not equidistant as for ordinary 2DEG but enumerated by $\sqrt{n}$, *n* is the number of the LL [23]. This form of the self-energies results from the linear in momentum Hamiltonian close to *K* and $K^{\prime }$ points, whereas the degeneracy of each LL subband is the same as for conventional 2DEG and equals to $\frac{BS}{hc/e}$. The number of subbands per each LL (each *n*) is here 4. This corresponds to the ordinary Zeeman spin-splitting and to the so-called valley splitting (expressed often in terms of a pseudospin) due to doublet of inequivalent *K* points mixed with two sublattices for *C* atoms in crystal lattice of graphene [23]. Taking into account that the Zeeman splitting in graphene is small [6] and the valley splitting depending on structure imperfections and the external magnetic field is small as well, the fourfold approximate spin-valley additional degeneracy is assumed to determine the filling fractions for ‘relativistic’ IQHE in graphene, in the following form $\nu =4(n+\frac{1}{2})$ in correspondence with the experimental observations [6]. The remarkable difference between this filling rate formula and that for the ordinary 2DEG (with factor 2 instead of 4, due to only approximate spin degeneracy) is the presence of the overall shift by the factor 2 ($\frac{1}{2}$ in the formula $\nu =4(n+\frac{1}{2})$). This shift is due to the Berry phase manifestation in the LLL for graphene and resulting in the sharing of the LLL states between particles and holes from the conduction and valence band at the zero energy level. Due to this feature the bottom of the LLL is shifted by 2 (in terms of the filling factor) upward if one counts only negatively charged carriers and oppositely – downward for valence band positive holes. The corresponding filling rates for holes from the valence band are a negative mirror refection of those for electrons from the conduction band. Note that changing between particles and holes can be easily achieved in graphene by the shift of the Fermi level around the Dirac point by application of a lateral voltage.

The Berry phase contribution is linked to an additional π phase shift due to the chiral valley pseudospin when one adiabatically traverses with a selected particle a closed loop (e.g. along the semiclassical cyclotron loop) with momentum 2π convolution.

Similar analysis of the LLs can be done for the bilayer graphene [23,24]. Due to off-diagonal interlayer hopping the local Hamiltonian attains back the quadratic form with respect to the momentum. Thus the LL spectrum in bilayer graphene resembles that for the ordinary 2DEG with four subbands for each level except for the LLL which is eightfold degenerated. This extra degeneracy of the LLL arises from the action of the square of an annihilation operator on oscillator states with n=0 and n=1 [23,24]. Because the LLL subbands are distributed among particles and holes, the bottom for uniformly charged carriers is located in the center of the eightfold quasi-degenerated LLL. This property is associated again with the Berry phase shift for chiral particles, though in the case of bilayer graphene it gives additional 2π phase shift. Therefore, steps of the relativistic IQHE are located in bilayer graphene back at integer filling rates of the subsequent LLs whereas for monolayer graphene were located at half-fillings of the LLs [23,24].

### 3.1. FQHE in monolayer graphene

For the magnetic field strong enough that ν∈(0,1) and by the lateral voltage Fermi level shifted to the conduction band, one deals with fractionally filled first conduction subband n=0,2↑ ( 2 indicates the valley pseudospin down-orientation and ↑ indicates orientation of the ordinary spin). The degeneracy of each subband is $N_{0}=\frac{BS}{hc/e}$ and for $N<N_{0}$ the filling rate, $\nu =N/N_{0}$, is fractional.

The cyclotron orbits in the LLL must be accommodated to the bare kinetic energy $T=\hbar \omega_{c}\left(n+\frac{1}{2}\right)$ with n=0, ($\omega_{c}=\frac{eB}{mc}$). The topology is not modified by the crystal field and to define the commensurability condition the braid cyclotron dimensions repeats those of the noninteracting gas. The cyclotron orbits have the same size for all particles due to the flat band quenching the kinetic energy competition. This results in the same averaged velocity and the same cyclotron orbit size for all particles in a perpendicular magnetic field (though in quantum mechanical treatment, velocity is not well defined since its coordinates do not commute). The cyclotron orbits restrict the braid topology of all trajectories uniformly in 2D, thus restrict the braid group structure despite particularities of the dynamics in the crystal field, which do not change trajectory topology. Therefore for graphene the cyclotron orbit structure is governed by ordinary (the same as for 2DEG) Landau levels restrictions despite the fact that a specific band structure with Dirac points, being the result of the crystal field in graphene, highly modifies LLs but not in terms of the bare kinetic energy. The specific for graphene quantum dynamics is included to the Feynman path integral, whereas the additional summation over topologically nonequivalent trajectory classes concerns the braid group structure the same as for 2DEG upon the magnetic field. The difference between the conventional 2DEG systems and graphene will be related in the regard to the statistics with distinct number of LL subbands in graphene compared to the conventional 2DEG and to the Barry phase shift of fillings in the LLL.

Thus, the cyclotron orbit size in the subband n=0,2↑ equals to $\frac{hc/e}{B}=\frac{S}{N_{0}}$ (*S* is the sample surface). As this orbit size is lower than the interparticle separation $\frac{S}{N}$ (as $N<N_{0}$), the multilooped braid structure is necessary. From the commensurability condition $q\frac{S}{N_{0}}=\frac{S}{N}$ one finds $\nu =\frac{N}{N_{0}}=\frac{1}{q}$, (q-oddinteger to protect the braid structure [9]). For holes in this subband one can expect the symmetric filling rates $\nu =1-\frac{1}{q}$. Similarly as for the ordinary 2DEG one can generalize this simple series by assumption that the last loop of the multilooped cyclotron orbit can be commensurate with the interparticle separation as for some other filling ratio expressed by l>1, whereas the former loops take away an integer number of flux quanta. For l=1,2,3,⋯ the last loop reaches every *l*-th particles. In this way one can obtain the hierarchy of fillings for FQHE in this subband of the LLL, $\nu =\frac{l}{l(q-1)\pm 1},\;\;\nu =1-\frac{l}{l(q-1)\pm 1}$, where l=1,2,⋯ and minus in the denominators indicates possibility of the eight-figure orientation of the last loop with respect to the antecedent one. The Hall metal states can be characterized by the limit l→∞ in the above formula (i.e., for zeroth flux taken away by the last loop, or equivalently, the last loops reaching infinitely distant particle, as for ordinary fermions without the magnetic field, which was the case in the Hall metal archetype for $\nu =\frac{1}{2}$ in the conventional 2DEG), which gives the hierarchy for the Hall metal states, $\nu =\frac{1}{q-1},\;\;\nu =1-\frac{1}{q-1}$. Some other possibility may correspond with the case when in *q*-looped orbit q-1 loops are accommodated to every *x*-th particle (x=1,2,3,⋯, whereas the last one fits to every *l*-th particle separation. Such an ordering is observed in ordinary 2DEG Hall systems in the LLL, for e.g. $\nu =\frac{4}{11},\frac{5}{13},\frac{3}{8},\frac{3}{10}$ (out of the CF hierarchy), but is not observed in the LLL in monolayer graphene to date, though it is observed in the first LL in monolayer graphene (as will be analyzed below). To account for the Berry phase anomaly in graphene the overall shift of ν by -2 can be performed, but we use here the net filling fractions.

For the completely filled subband n=0,2↑, i.e., for ν=1, one arrives at IQHE. For a lower magnetic field (or a larger number of electrons), when the three first subbands (two of them belonging to valence band holes) of the LLL are filled and in the last subband of LLL n=0,2↓, the cyclotron orbit size $\frac{S}{N_{0}}$ is still lower than the interparticle separation $\frac{S}{N-N_{0}}$ (because $N-N_{0}<N_{0}$). Thus multilooped structure is repeated from the previous subband. This results with the same FQHE hierarchy as for antecedent subband, only shifted ahead by 1. For the completely filled LLL (i.e., for all four of its subbands completely filled) one deals with IQHE according to its main-line $\nu =4(n+\frac{1}{2})$.

Similarly one can consider fillings of the following LL with n=1. This level also has four subbands, but in this level the bare kinetic energy is equal to $\frac{3\hbar \omega_{c}}{2}$ and the related cyclotron orbit size is $\frac{3hc}{eB}=\frac{3S}{N_{0}}$. For $N\in (2N_{0},3N_{0}]$ we deal with graduate filling of n=1,1↑ subband. Cyclotron orbits of size $\frac{3S}{N_{0}}$ must be compared here with interparticle separation scale $\frac{S}{N-2N_{0}}$. For a small number of electrons in this subband one deals with the multilooped structure (corresponding to the inequality $\frac{3S}{N_{0}}<\frac{S}{N-2N_{0}}$), when $q\frac{3S}{N_{0}}=\frac{S}{N-2N_{0}},\;q-odd\;integer$, which gives the main series for FQHE(multiloop) in this subband, $\nu =2+\frac{1}{3q}$. Similarly as before the complete related hierarchy can be written as $\nu =2+\frac{l}{l3(q-1)\pm 1},\;\;\nu =3-\frac{l}{l3(q-1)\pm 1}\;l=i/3,\;i=1,2,\cdots$, with the Hall metal hierarchy in the limit l→∞. These series are located closer to the subband edges, whereas in the center of this subband the other commensurability conditions are possible. When $\frac{3S}{N_{0}}=\frac{xS}{N-2N_{0}}$ and x=1,2,3 (fitting of the single-looped orbit with every *x*-th particle) we get $\nu =\frac{7}{3},\frac{8}{3},3$, respectively, corresponding to single-looped cyclotron orbits similar as for IQHE. Thus, for $\nu =\frac{7}{3},\frac{8}{3}$ one deals with FQHE(single-loop) – the new Hall feature manifesting itself only in higher LLs, where cyclotron orbits may be larger than the interparticle separation.

At the special case $\frac{3S}{N_{0}}=\frac{1.5S}{N-2N_{0}}$ one can arrive at $\nu =\frac{5}{2}$ with the paired particles (pairing does not change the cyclotron radius but twice diminishes the carrier number to $\frac{N-2N_{0}}{2}$, which gives the commensurability for pairs at $\nu =\frac{5}{2}$).

Moreover, for *q*-looped orbits, their size may be accommodated to every *x*-th particle in the subband, resulting in additional hierarchy in all subbands of the first LL, $\nu =2\left(3,4,5\right)+\frac{xl}{l3(q-1)\pm 1}$, $\nu =3\left(4,5,6\right)-\frac{xl}{l3(q-1)\pm 1}$ which for q=3,x=2,3,l=i/3,i=1,2,3 reproduces $\nu =\frac{7}{3},\frac{8}{3},\frac{12}{5},\frac{13}{5},\frac{17}{7},\frac{18}{7},\frac{22}{9},\frac{23}{9},\frac{10}{3},\frac{11}{3},\frac{17}{5},\frac{18}{5},\frac{24}{7},\frac{25}{7},\frac{13}{3},\frac{14}{3},\frac{22}{5},\frac{23}{5}$ with FQHE features recently observed in the three first subbands of the n=1 LL in monolayer graphene at ultra-low temperatures [5].

The following subbands are filled with electrons upon the similar scheme. For the subband n=1,1↓ the cyclotron orbit size is $\frac{3hc}{eB}=\frac{3S}{N_{0}}$ (as in all subbands with the same *n*), whereas the interparticle distances are measured with the plaque $\frac{S}{N-3N_{0}}$, where $N\in (3N_{0},4N_{0})$. The commensurability condition $\frac{q3S}{N_{0}}=\frac{S}{N-3N_{0}}$ results in the main series for FQHE(multiloop), $\nu =3+\frac{1}{3q}$, which can be developed to the full hierarchy similarly as described above. The condition $\frac{3S}{N_{0}}=\frac{xS}{N-3N_{0}}$ with x=1,2,3 results in fractions with single-looped correlations of FQHE(single-loop)-type for $\nu =\frac{10}{3},\frac{11}{3}$ and IQHE for ν=4, correspondingly, whereas a paired state can be realized at $\nu =\frac{7}{2}$.

### 3.2. FQHE in bilayer graphene

The special topology of the bilayer graphene creates the opportunity to verify the braid group based concept of the commensurability of cyclotron orbit size with the interparticle spacing in the planar system of interacting particles. Let us emphasize once more that the interaction is essential for correlation manifestation – the interaction prevents particles from approaching one another closer than the particle separation resulting from the planar density. This separation is rigidly kept by the interaction allowing the commensurability notion to be useful. Exclusively when the cyclotron orbits fit accurately to the particle spacing the exchange of neighboring particles in the presence of the perpendicular magnetic field is possible along specific braids named as the cyclotron braids.

It must be emphasized that commensurability condition, as related to the braid group, concerns the classical archetype of the quantum system, thus is not addressed to real quantum particles which do not have any trajectories. This agrees with the spirit of the braid group approach in which classical trajectories in configuration space supply information on quantum statistics via 1DURs of the braid group. To organize any correlated collective state, the related statistics of particles must be determined first. In order to establish the statistics of quantum particles, the appropriate braid group must be defined whereas the statistics is governed by the selected 1DUR of this group [13].

The bilayer graphene is, however, not strictly two dimensional and for the bilayer graphene the topological situation changes considerably. Two sheets of the graphene plane lie close to each other and the hopping can change the electron positions among the planes. Thus, we deal here with double the number of electrons residing on a two-sheet structure simultaneously instead of the single sheet as was the case for the monolayer graphene.

The above requirements, to fulfill the commensurability condition in order to define the related braid group describing the correlated multiparticle state, also apply to the bilayer graphene, with a single distinction with respect to the monolayer case. The *doublelooped* cyclotron orbits may have in bilayer graphene the same size as the single-looped orbit in opposition to the monolayer case. This follows from the fact that the second loop can be located in the opposite sheet of graphene than the first one and the external field passing through such doublelooped orbit is twice larger than the flux passing through the single-looped orbit. Each loop has in this case its own separate surface in contrary to the multilooped cyclotron orbit located on the purely 2D plane. Taking into account that in the bilayer system loops of the multilooped orbit may be located partly in both 2D sheets, the contribution of the one loop must be avoided whereas the remaining loops must share the same flux as passing through a single-looped orbit, independently of how loops are distributed among two sheets. Thus, one can write out the commensurability condition in the bilayer graphene for the case of too short single-looped cyclotron orbits in the following form (for concreteness in the subband n=0,2↑ of the LLL – the first particle-type subband of the LLL):

(3) $$\begin{array}{c} \begin{array}{c} \frac{hc}{eB}=\frac{S}{N_{0}}<\frac{S}{N},\\ \left(p-1\right)\frac{hc}{eB}=\left(p-1\right)\frac{S}{N_{0}}=\frac{S}{N},\\ \nu =\frac{N}{N_{0}}=\frac{1}{p-1}=\frac{1}{2},\frac{1}{4},\frac{1}{6},\cdots , \end{array} \end{array}$$

where, *N* is the total number of particles in both graphene sheets, $N_{0}$ is the degeneracy counted for both sheets together, *S* is the surface of the sample (the surface of the single sheet), and *p* is an odd integer to assure that the half of the cyclotron orbit defines the braid.

The factor p-1 in Equation (3) is caused by the fact that only orbits from the ideal 2D sheets of bilayer graphene contribute to the enlargement of the effective cyclotron orbits (no matter in which are located doubling loops) with exception of a single orbit which may be located in the opposite sheet to the first one. This sole loop contributes to the total flux with the additional flux quantum due to its own surface and this loop must be omitted. The next orbits must duplicate the former ones (in fact two) without rising to the surface and no matter in which sheet are they located, because in both they will duplicate loops already present there. Thus, in the enhancement of the effective *p*-looped cyclotron orbit take part only p-1 loops.

**Figure 4.**
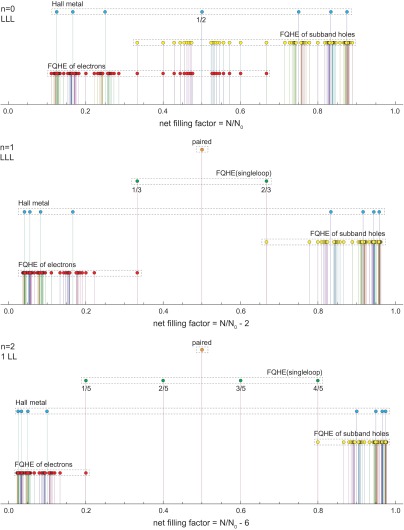
Evolution of fractional filling hierarchy in three first LLs of the monolayer graphene; for each LL the first particle subband is illustrated, the next subbands in each LL repeat the hierarchy from the first one. Different types of ordering are indicated with spikes of various height. Series for ordinary FQHE(multiloop), FQHE(single-loop), Hall metal and paired state are displayed according to the hierarchy described in Table 1 with q=3-9, 1=1-10; only a few selected ratios from these series are explicitly written out.

Let us emphasize that for such multilooped orbits the total number of loops is still *p* – thus the generators of the corresponding cyclotron subgroup are of the form $b_{i}^{\left(p\right)}=\sigma_{i}^{p}$, resulting in the Laughlin correlations with the *p* exponent for the Jastrow polynomial. But due to the distinct commensurability of orbits with interparticle separation (Equation (3)) the related filling fractions are $\nu =\frac{1}{p-1}$ (*p*-odd) in the first particle-type subband of the LLL, i.e., in the subband n=0,2↑. This even denominator main series of the FQHE hierarchy for bilayer graphene pretty well coincides with the experimental observations [1].

For holes in this subband (let us emphasize that these holes are not holes from the valence band but correspond to unfilled states in the almost filled subband of particle type) one can write $\nu =1-\frac{1}{p-1}$, whereas the generalization to the full hierarchy of FQHE in this subband attains the form, $\nu =\frac{l}{l(p-2)\pm 1},\;\;\nu =1-\frac{l}{l(p-2)\pm 1}$, where l>1 corresponds to some filling factor for other correlated Hall state, including completely filled LLs with IQHE. In the next subbands of the LLL, n=0,2↓ (assuming that this subband succeeds the former one), the hierarchy is identical only shifted ahead by one, because commensurability condition has the same form for all subbands with the same *n* due to the same size of the cyclotron orbits.

Some novelty occurs, however, in the following two subbands of the LLL, n=1,2↑ and n=1,2↓. The FQHE main series in the first of these subbands of the LLL, n=1,2↑, has the form,

(4) $$\begin{array}{c} \begin{array}{c} \frac{3hc}{eB}=\frac{3S}{N_{0}}<\frac{S}{N-2N_{0}},\\ \left(p-1\right)3\frac{hc}{eB}=\left(p-1\right)\frac{3S}{N_{0}}=\frac{S}{N-2N_{0}},\\ \nu =\frac{N}{N_{0}}=2+\frac{1}{3(p-1)}=2+\frac{1}{6},2+\frac{1}{12},2+\frac{1}{18},\cdots , \end{array} \end{array}$$

The generalization of this main series for holes in the subband and to the full FQHE hierarchy in this subband is as follows: for subband holes, $\nu =3-\frac{1}{3(p-1)}$ and for the full FQHE hierarchy in this subband, $\nu =2+\frac{l}{l3(p-2)\pm 1},\;\;\nu =3-\frac{l}{l3(p-2)\pm 1},\;l=\frac{i}{3},\;i=1,2,3,\cdots$ (with Hall metal hierarchy in the limit l→∞).

**Table 1.** LL filling factors for FQHE determined by commensurability condition (*paired* indicates condensate of electron pairs), for the first particle subband in each of the three first LLs (n=0,1,2) for the monolayer graphene.; subb. stands for subband.

LL subb.	FQHE(single-loop), paired, IQHE	FQHE(multiloop) (q-odd, $l=\frac{i}{2n+1}$, i=1,2,3,⋯)	Hall metal
n=0,2↑	1	$\frac{1}{q},1-\frac{1}{q},\frac{l}{l(q-1)\pm 1},1-\frac{l}{l(q-1)\pm 1}$	$\frac{1}{q-1},1-\frac{1}{q-1}$
n=1,1↑	$\frac{7}{3}$, $\frac{8}{3}$, ($\frac{5}{2}\;paired$), 2, 3	$2+\frac{1}{3q}$, $3-\frac{1}{3q}$, $2+\frac{l}{3l(q-1)\pm 1}$, $3-\frac{l}{3l(q-1)\pm 1}$	$2+\frac{1}{3(q-1)}$, $3-\frac{1}{3(q-1)}$
n=2,1↑	$\frac{31}{5}$, $\frac{32}{5}$, $\frac{33}{5}$, $\frac{34}{5}$, ($\frac{13}{2}\;paired$), 6, 7	$6+\frac{1}{5q}$, $7-\frac{1}{5q}$, $6+\frac{l}{5l(q-1)\pm 1}$, $7-\frac{l}{5l(q-1)\pm 1}$	$6+\frac{1}{5(q-1)}$, $7-\frac{1}{5(q-1)}$

**Table 2.** LL filling factors for FQHE determined by commensurability condition (*paired* indicates condensate of electron pairs), for the first particle subband in each of the two first LLs (n=0,1 for the extra degenerated LLL and n=2 for the first LL beyond the LLL) for the bilayer graphene.

LL subb.	FQHE(single-loop), paired, IQHE	FQHE(multiloop) (q-odd, $l=\frac{i}{2n+1}$, i=1,2,3,⋯)	Hall metal
n=0,2↑ (LLL)	1	$\frac{1}{\left(q-1\right)}$, $1-\frac{1}{\left(q-1\right)}$, $\frac{l}{l(q-2)\pm 1}$, $1-\frac{l}{l(q-2)\pm 1}$	$\frac{1}{q-2}$,$1-\frac{1}{q-2}$
n=1,2↑ (LLL)	$\frac{7}{3}$, $\frac{8}{3}$, ($\frac{5}{2}\;paired$), 2, 3	$2+\frac{1}{3(q-1)}$, $3-\frac{1}{3(q-1)}$, $2+\frac{l}{3l(q-2)\pm 1}$, $3-\frac{l}{3l(q-2)\pm 1}$	$2+\frac{1}{3(q-2)}$, $3-\frac{1}{3(q-2)}$
n=2,1↑ (first LL)	$\frac{21}{5}$, $\frac{22}{5}$, $\frac{23}{5}$, $\frac{24}{5}$, ($\frac{9}{2}\;paired$), 4, 5	$4+\frac{1}{5(q-1)}$, $4+\frac{l}{5l(q-2)\pm 1}$, $5-\frac{1}{5(q-1)}$, $5-\frac{l}{5l(q-2)\pm 1}$	$4+\frac{1}{5(q-2)}$, $5-\frac{1}{5(q-2)}$

**Table 3.** Comparison of filling hierarchy in the LLL level in the bilayer graphene for two mutually inverted successions of two lowest subbands: n=0,2↑, n=1,2,↑ (upper) and n=1,2↑, n=0,2↑ (lower).

LL subb.	FQHE(single-loop), paired, IQHE	FQHE(multiloop) (q-odd, $l=\frac{i}{2n+1}$, i=1,2,3,⋯)	Hall metal
n=0,2↑	1	$\frac{1}{\left(q-1\right)}$, $1-\frac{1}{\left(q-1\right)}$, $\frac{l}{l(q-2)\pm 1}$, $1-\frac{l}{l(q-2)\pm 1}$	$\frac{1}{q-2}$, $1-\frac{1}{q-2}$
n=1,2↑	$\frac{4}{3}$, $\frac{5}{3}$, ($\frac{3}{2}\;paired$), 1, 2	$1+\frac{1}{3(q-1)}$, $2-\frac{1}{3(q-1)}$, $1+\frac{l}{3l(q-2)\pm 1}$, $2-\frac{l}{3l(q-2)\pm 1}$	$1+\frac{1}{3(q-2)}$, $2-\frac{1}{3(q-2)}$
n=1,2↑	$\frac{1}{3}$, $\frac{2}{3}$, ($\frac{1}{2}\;paired$), 1	$\frac{1}{3(q-1)}$, $1-\frac{1}{3(q-1)}$, $\frac{l}{3l(q-2)\pm 1}$, $1-\frac{l}{3l(q-2)\pm 1}$	$\frac{1}{3(q-2)}$, $1-\frac{1}{3(q-2)}$
n=0,2↑	1,2	$1+\frac{1}{q-1}$, $1+\frac{l}{l(q-1)\pm 1}$, $2-\frac{1}{q-1}$, $2-\frac{l}{l(q-2)\pm 1}$	$1+\frac{1}{q-2}$, $2-\frac{1}{q-2}$

In the subband n=1,2↑ of the LLL the new commensurability opportunity occurs (the one which appeared only in the first LL of the monolayer graphene): $\frac{3}{N_{0}}=\frac{x}{N-2N_{0}}$ for x=1,2,3, which gives fillings ratios $\nu =\frac{7}{3},\frac{8}{3},3$, correspondingly. All these rates are related with single-looped cyclotron trajectories, thus with single-loop correlations similar as for IQHE (though the first two for not integer filling rates). This new Hall feature, typical for LLs with n≥1, we called FQHE(single-loop). Moreover, for x=1.5 one can consider twice diminishing of particle number $(N-2N_{0})/2$ due to the pairing, which gives perfect commensurability of cyclotron orbits of pairs with the separation of the particle pairs at $\nu =\frac{5}{2}$.

The last subband n=1,2↓ in the LLL in bilayer graphene is filled with electrons in the similar manner because for both subbands with n=1 the cyclotron orbits have the same size. Thus the hierarchy of fractional filling for the last subband in the LLL is shifted by 1 from the antecedent subband without any modification. The situation changes, however, in the next LL (the first one beyond the LLL). In the first such LL (with n=2) the cyclotron orbits suited to commensurability condition are determined by the bare kinetic energy for n=2, and the corresponding cyclotron orbit size is equal to $\frac{5hc}{eB}=\frac{5S}{N_{0}}$. The similar analysis as in the previous LL gives here the main series and the full hierarchy for FQHE(multiloop) in the subband n=2,1↑, $\nu =4+\frac{1}{5(p-1)}$, $\nu =4+\frac{l}{l5(p-2)\pm 1},\;l=\frac{i}{5},\;i>1$, respectively (inclusion of subband holes resolves itself to the substitution of 4+ by 5- in both above formulae). Similarly as previously, the limit l→∞ gives the Hall metal hierarchy. The difference in comparison to the previous LL consists here also in the presence of four (instead two) satellite FQHE(single-loop) states symmetrically located around the central paired state. In the subband n=2,1,↑ the satellite states occur at $\nu =\frac{21}{5},\frac{22}{5},\frac{23}{5},\frac{24}{5}$ and the central paired state at $\nu =\frac{9}{2}$. This hierarchy scheme is repeated in all four subbands of the first LL.

**Figure 5.**
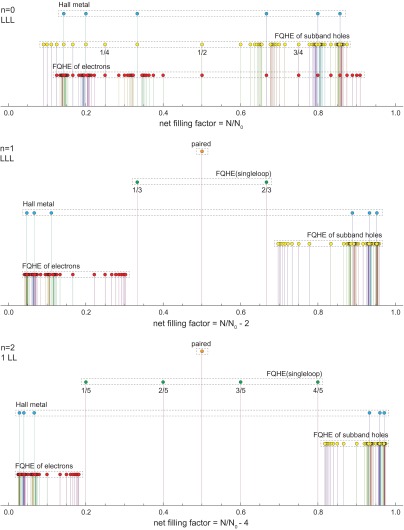
Evolution of fractional filling hierarchy in the two first LLs of the bilayer graphene; for the LLL two subbands with n=0 and n=1 are illustrated. Different types of ordering are indicated with spikes of various heights. Series for ordinary FQHE(multiloop), FQHE(single-loop), Hall metal and paired state are displayed according to the hierarchy described in Table 2 with q=3-9, i=1-10; only a few selected ratios from these series are explicitly written out.

The evolution of the fractional filling hierarchy of subsequent LLs is illustrated in Figures 4 and 5, for the monolayer and bilayer graphene, correspondingly and is summarized in Tables 1 and 2.

For bilayer graphene the degeneration of n=0 and n=1 states results in eightfold degeneracy of the LLL, doubling fourfold spin-valley degeneracy. The degeneracy is not exact and with rising magnetic field amplitude both the Zeeman splitting and the valley splitting grows. Stress, deformation and structure imperfections also cause the increase of the valley splitting. Inclusion of the interaction plays a similar role. Coulomb interaction causes mixing of n=0,1 states lifting their degeneracy. Especially interesting is such a degeneracy lifting which admits inverted order of fillings of LLL subbands with distinct n=0,1. The inversion of orders n=0,1 to n=1,0 affects the filling rates hierarchy. Assuming that the LLL subband with n=1 is filled earlier than the n=0 subband, we get the following hierarchy for the first subband n=1,2↑: multilooped orbits for $\nu =\frac{l}{l3(p-2)\pm 1}$, $\nu =1-\frac{l}{l3(p-2)\pm 1}$, single-looped orbits for $\nu =\frac{1}{3},\frac{2}{3}$ and a paired state for $\nu =\frac{1}{2}$. Assuming the next subband, n=0,2↑, we get the hierarchy of fillings for this subband in the form: multilooped orbits for $\nu =1+\frac{l}{l(p-2)\pm 1}$, $\nu =2-\frac{l}{l(p-2)\pm 1}$ and no single-looped orbits. The comparison of reverted orderings of two first LLL subbands is summarized in Table 3.

One can consider also the situation in the LLL of bilayer graphene, when the degeneracy of n=0,1 states is lifted is such a way that both levels cross at certain filling factor $\nu^{*}<1$ (cf. [25], where mixing between n=0,1 states is numerically analyzed for small models on torus or sphere). Let us assume for a model that first the n=1 subband is filled (n=1,2↑) up to $\nu^{*}$. At this filling the subband n=1,2↑ crosses with the subband n=0,2↑ and the latter is filled for $1+\nu^{*}>\nu >\nu^{*}$. The related hierarchy of fractional fillings looks like an ordinary filling of the subband n=1,2↑, however, with an insertion of n=0,2↑ filling structure. Depending on the value of $\nu^{*}$ the various patterns are achievable by simple combination of hierarchy patterns listed in Table 3 (including also inverted ordering of n=1 and n=0 subbands).

## 4. Comparison with experiment

**Figure 6.**
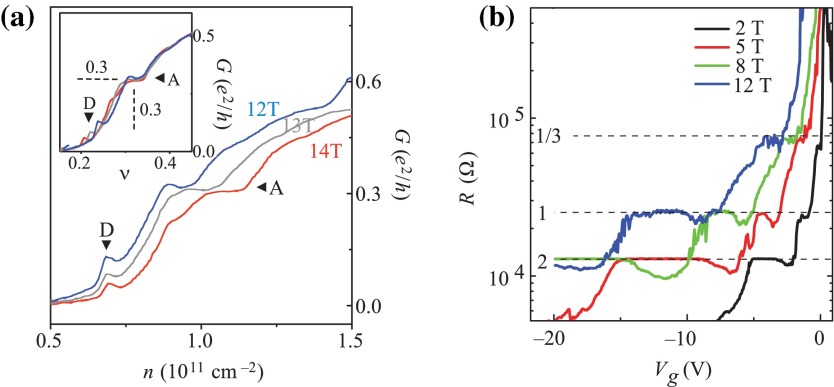
(a) FQHE observation in suspended graphene for the filling 0.3 (1 / 3) in a field of 12–14 T with the concentration of $10^{11}$ cm${}^{-2}$ and the mobility of 250,000 cm${}^{2}$V${}^{-1}$s${}^{-1}$; (b) FQHE singularities in suspended graphene for the filling $\frac{1}{3}$ in a field of 2–12 T with the concentration of $10^{10}$ cm${}^{-2}$ and the mobility of 200,000 cm${}^{2}$V${}^{-1}$s${}^{-1}$ (after [28,29]).

Searching for FQHE states in graphene is particularly challenging because of the different ‘relativistic’ structure of LLs, which is more complicated than the conventional semiconductor 2DEG. Moreover, the filling factor can be changed in graphene both by the external magnetic field and by the particle concentration via shifting of the Fermi level near the Dirac points by application of the lateral voltage. Due to spin-valley degeneracy and Berry phase contribution related to the chiral valley pseudospin, the IQHE is observed in graphene for fillings $\nu =4(n+\frac{1}{2})=2,6,10,14,\cdots$ for particles from the conduction band and for the mirror negative factors for holes from the valence band. Despite using very strong magnetic fields (up to 45 T), FQHE was, however, not detected in graphene samples deposited on a substrate of $SiO_{2}$ [7]. Instead, at these strong magnetic fields the emergence of additional plateaus of IQHE has been observed for the fillings ν=0,±1,±4, indicating spin-valley degeneracy lifting as a result of an increase of the mass of Dirac fermions [7]. Only after mastering the technology of the so-called suspended ultrasmall graphene scrapings with extreme purity and high mobility of carriers above 200,000 cm${}^{2}$V${}^{-1}$s${}^{-1}$ (high mobility is necessary to observe FQHE also in the case of conventional semiconductor 2D heterostructures, which may be related to multi-looped quasi-classical cyclotron movement of wave packets in the case of multi-looped braids associated with FQHE [26]; note that in conventional semiconductor 2D heterostructures carrier mobility reaches even higher values of millions cm${}^{2}$V${}^{-1}$s${}^{-1}$ [27]), it was possible to observe FQHE in graphene at net fillings ν=1/3 and -1/3 (the latter for holes, at the opposite polarization of the gate voltage, which determines position of the Fermi level, either in the conduction band, or in the valence band) [28,29]. Both these papers report the observation of FQHE in graphene for medium strong magnetic fields: in a field of 14 T, for electron concentration of $10^{11}$ cm${}^{-2}$ [28]; and in a field of 2 T, but for a concentration level smaller by one order of magnitude ($10^{10}$ cm${}^{-2}$ and the mobility of 200,000 cm${}^{2}$V${}^{-1}$s${}^{-1}$) [29], as shown in Figure 6.

FQHE in suspended graphene is observed at relatively high temperatures around 10 K [30], and even higher (up to 20 K) [31], which seems to be related with the stronger electric interaction in view of the lack, in the case of suspended samples, of a dielectric substrate (with the dielectric constant in case of $SiO_{2}$, ∼3.9) and, on the other hand, with very high cyclotron energy in graphene (i.e., large energy gap between incompressible states).

**Figure 7.**
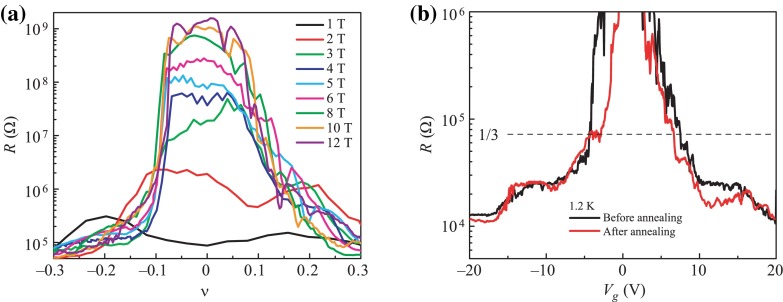
The emergence of an insulator state accompanying the increase in the strength of a magnetic field around the Dirac point; (b) competition between FQHE and the insulator state for the filling -1/3: annealing removes pollution – enhances mobility and provides conditions for the emergence of *plateau* for FQHE (after [29]).

The competition between the FQHE state with the insulator state near the Dirac point, corresponding to a rapidly decreasing concentration has also been demonstrated [32,33] (Figure 7).

From the perspective of cyclotron groups, experimental results on FQHE in graphene [28–31,34] seem to be compliant with the expectations of the braid description. In the case of graphene, the specific band structure with conical Dirac bands leads to simultaneous participation (in Dirac point) of both bands – of holes and of electrons, which combined with the massless character of Dirac fermions manifests itself through an anomalous ‘relativistic’ IQHE [7,32,35]. Controlling lateral gate voltage (within the range ca. 5-60 V [1,4,28]) allows regulation of the density of carriers at a constant magnetic field. One should therefore expect that at relatively small densities of carriers (electrons, or symmetrical holes at reverse voltage polarization), the cyclotron orbits will be too short to admit braid exchanges of particles at a sufficiently strong magnetic field – although weaker for smaller concentrations – and experimental observations exactly support this [28,29]. For low concentration, while closing on the Dirac point, one may expect that too strong fields would exceed the stability threshold of the FQHE state in competition with the Wigner crystal (assuming a similar character of this competition in the case of massless Dirac fermions in reference to conventional semiconductor 2D structures) and that it corresponds to the emergence of the insulating state near the Dirac point in a sufficiently large magnetic field as visible in experiment [36]. In the case of the hexagonal structure of graphene, electron (or hole) Wigner crystallization [37] may exhibit interference between the triangular crystal sublattices, and including of the resonance (hopping) between these two sublattices may cause blurring of the sharp transition to the insulator state, which seems compliant with observations (Figure 7).

The progress in the experiment allowed also for observation of FQHE in graphene on the crystal substrate of boron nitride (*BN*) in high magnetic fields of the order of 40 T (remarkably, FQHE features were noticed in this case up to ν=4) [4,5].

The mobility of carriers in graphene is lower than in traditional 2DEG, but taking into account that the carrier concentration in graphene can be lower in comparison to semiconductor heterostructure [27,38], the corresponding mean free path in both cases well exceeds the sample dimension (of μm order, as the mobility is proportional to the concentration and to the mean free path of carriers).

Energy gaps protecting incompressible FQHE states are larger in graphene than in traditional semiconductor materials, reaching a magnitude in order of 16 K (at $\nu =\frac{4}{3}$ and B=35 T), which is referred to Dirac massless character of carriers. In conventional semiconductor heterostructure the corresponding gaps are much lower and the observed FQHE stability with temperature is much more fragile.

**Figure 8.**
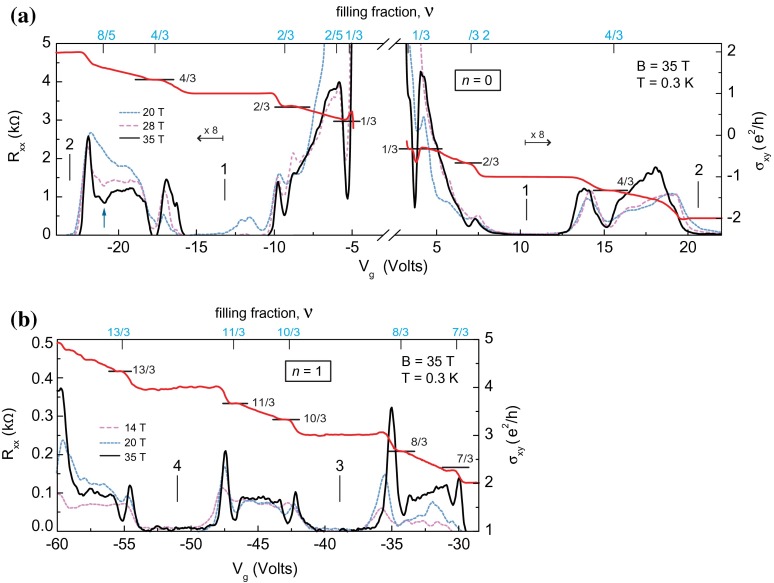
Fractional quantum Hall effect for graphene on BN. Magnetoresistance (left axis) and Hall conductivity (right axis) in the n=0 and n=1 Landau levels at B=35 T and temperature ∼0.3 K (after [4]). All filling ratios indicated in blue agree with the hierarchy given in Table 1.

**Figure 9.**
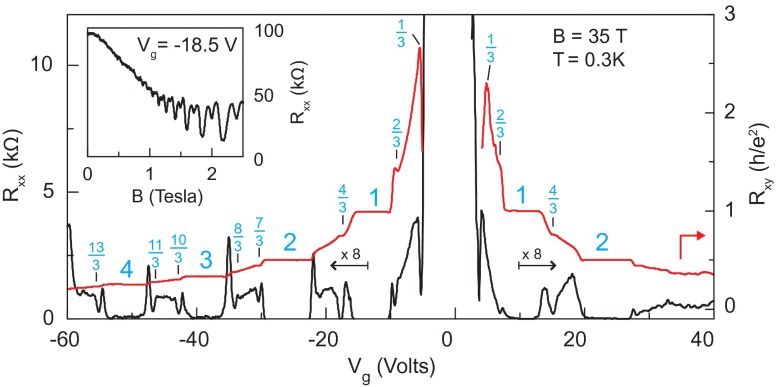
Magnetoresistance (left axis) and Hall resistance for graphene on BN (right axis) versus gate voltage acquired at B = 35 T. Inset shows Shubnikov-de Haas oscillations at Vg = –18.5 V (after [4]). All filling ratios indicated in the figure (in blue) agree with the hierarchy given in Table 1.

The recent development in experiments with monolayer graphene on BN substrate [4,5] and with suspended small sheets [2,3] allowed for observation of more and more Hall features at fractional fillings of subsequent subbands of two first LLs. While the sequence of fillings in the lowest subband of the LLL fits well to CF predictions (including CFs with two and four flux quanta attached), an explanation of the filling structure of next subbands strongly deviates from this simple picture. The pattern of filling rates repeated in the subbands of the first LL goes beyond the CF concept [2,3,5]. This phenomenon is referred in these papers to the various scenarios of breaking of the approximate SU(4) spin-valley symmetry in graphene. Because of the smallness of the Zeeman splitting $E_{Z}$ in comparison to the Coulomb energy in graphene, $E_{Z}/E_{C}∼0.01\varepsilon$, similarly as of lattice scale in comparison to the magnetic length, $a/l_{B}∼0.06$ (where $l_{B}=\sqrt{\frac{h}{eB}}$ and ε is the dielectric susceptibility ∼3.2) [4], the subbands which differ with spin and valley-pseudospin orientation are closely located and can be regarded as approximately degenerated. This SU(4) spin-valley symmetry can be next broken by various factors and one can search arguments for unusual filling ratios hierarchy in related symmetry breaking and phase-like transitions. Despite there being many related ideas, no fully consistent picture has yet been attained in this way.

**Figure 10.**
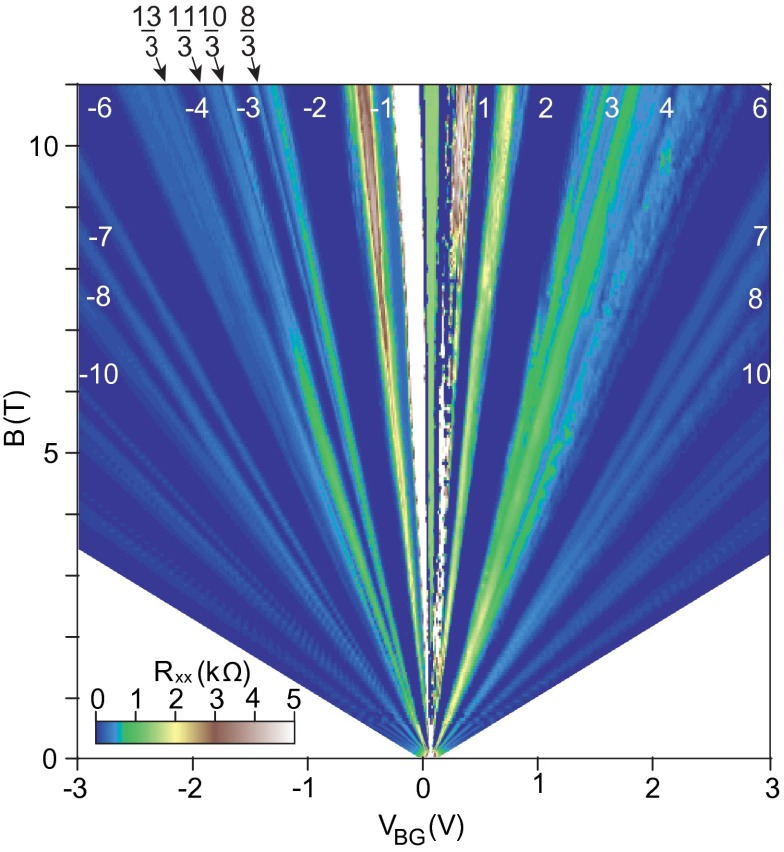
Fan diagram for $\rho_{xx}\left(\nu ,B\right)$ for graphene up to 11 T (after [5]).

**Figure 11.**
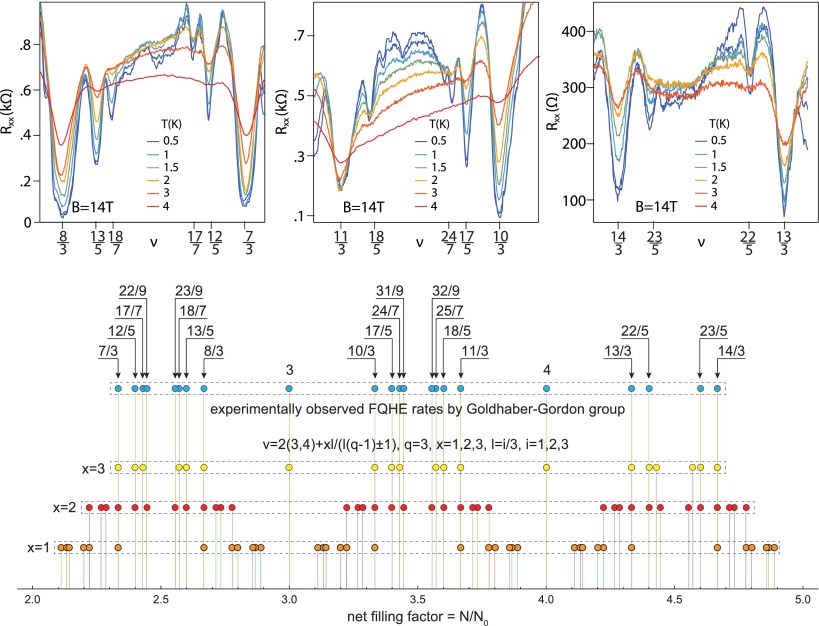
Not fully developed FQHE states with residual longitudinal resistance, corresponding to correlation of every second or every third particles at fractional rates reproduced by the commensurability series $\nu =2\left(3,4\right)+\frac{xl}{l3(q-1)\pm 1}$ with $q=3,\;x=2,3,l=\frac{i}{3},\;i=1,2,3$ (upper panels visualize longitudinal resistivity measurements after [5]).

**Figure 12.**
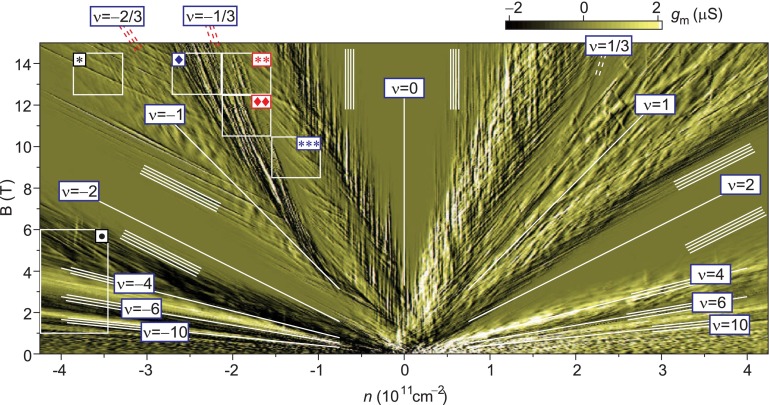
Color rendition of the transconductance in (*N*, *B*) plane – the tiny pattern agrees with the hierarchy for monolayer graphene given in Table 1 (after [39]).

If one compares the experimentally observed fractions for characteristic FQHE features in longitudinal and Hall conductivities measured on a variety of samples of graphene with the pattern of fractional hierarchy for two lowest LLs, as illustrated in Figure 4, one notices the coincidence of this hierarchy with the measured data. All fractions found experimentally can be reproduced by this hierarchy (cf. Table 1). From this comparison it is visible why the CFs are efficient only in the LLL. This is linked with the fact that, exclusively in the LLL, cyclotron orbits are always shorter than the interparticle separation and additional loops are necessary. These loops can be modeled by fictitious field flux quanta attached to CFs. Though the analogy of CF flux quanta to additional loops is not exact, it allows one to get at least the similar main line of the filling hierarchy in the LLL as that given by the commensurability condition. The usefulness of the CF model is, however, limited in higher LLs because beginning from the first LL the multilooped commensurability is needed rather close to the subband edges, whereas the central regions of all subbands of the first LL are occupied by the following doublets of filling factors: ($\frac{7}{3},\frac{8}{3}$), ($\frac{10}{3},\frac{11}{3}$), ($\frac{13}{3},\frac{14}{3}$), ($\frac{16}{3},\frac{17}{3}$), corresponding to single-looped commensurability condition, not related to CF modeling, but visible in experiments as FQHE(single-loop) [2–5]. The number of centrally located filling rates for FQHE(single-loop) grows next with the LL number as 2*n*. The repeating doublet of filling ratios for n=1 is noticeable in Figure 9 and in more accurate measurements in suspended samples [2,3] besides of those on the BN substrate [4,5]. Worth noting is the observation [5] that stability of FQHE(single-loop) states is of similar order as of IQHE states and higher in comparison to FQHE(multiloop) state features as is visible in Figure 10. This might be associated with stronger correlations related to the single-looped braids as for IQHE states.

The hierarchy induced by the commensurability condition reproduces the positions also of other observed features in two lowest LLs in the case of the monolayer graphene. The elongate plateaus at edges of subbands, with IQHE-rates in the centers, embrace minima related to closely located FQHE(multiloop)-rates indistinguishable at the observation resolution. In higher LL the new features, however, occur in between the above-mentioned doublets, but with nonzero longitudinal resistivity contrary to other FQHE states. This suggests that not all particles participate in these correlated states and they may correspond to the multilooped correlations of every second or every third particles according the appropriate cyclotron commensurability. And indeed these new features in the first LL of monolayer graphene, recently reported [5] at $\nu =\frac{7}{3},\frac{8}{3},\frac{12}{5},\frac{13}{5},\frac{17}{7},\frac{18}{7},\frac{22}{9},\frac{23}{9},\frac{10}{3},\frac{11}{3},\frac{17}{5},\frac{18}{5},\frac{24}{7},\frac{25}{7},\frac{13}{3},\frac{14}{3},\frac{22}{5},$-pagination

$\frac{23}{5}$, are reproduced one-to-one by the commensurability series $\nu =2\left(3,4\right)+\frac{xl}{l3(q-1)\pm 1}$ with $q=3,\;x=2,3,\;l=\frac{i}{3},\;j=1,2,3$ as shown in Figure 11. One can notice that the rates $\frac{7}{3},\frac{8}{3},\frac{10}{3},\frac{11}{3},\frac{13}{3},\frac{14}{3}$ are repeated in this hierarchy, though they correspond to single-looped more stable correlation as is visible in Figure 10 and in the upper panel of Figure 11.

**Figure 13.**
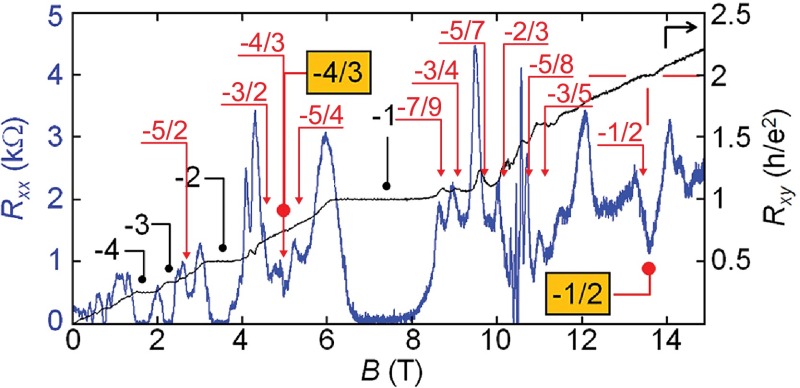
Observation of FQHE at T = 0.25 K in bilayer suspended graphene. Magneto-resistance Rxx (blue curve) and Rxy (black curve) at the lateral voltage –27 V (after [1]). Red symbols represent ratios from the hierarchy given in Table 2.

Another experimental evidence for the above hierarchy follows from the measurement [39] of the transconductance in the single electron field effect transistor configuration of small scrapings of graphene (0.8×3μm) in a varying gate voltage $V_{bg}+\delta V_{bg}\left(t\right)$ (f=433 Hz, $V_{bg}∼1$ V, $\delta V_{bg}∼10$ mV), which has revealed a fine structure of local correlated states. This structure is visible in typical transport experiments as noise-like oscillations but in the not-noisy regime. The detailed inspection of this fluctuations was done by visualization of the transconductance in the (*N*, *B*)-plane in Figure 12, which revealed a highly ordered pattern attributed to series of local correlated states closely accompanying fractions for the IQHE (and the FQHE as well) in monolayer graphene. The linear character of these new features was discovered, including several bunches collinear to directions of main IQHE/FQHE ratios in the (*N*, *B*)-plane. Straight lines, which lie in a (*N*, *B*)-plane and have a common point in a coordinate origin, are connected to a constant filling factor value; $B=\frac{hc}{e\nu }N$ (units are selected that $\frac{hc}{e}=1$). An arbitrary line in a (*N*, *B*)-plane can be described as B=αN+β. Its successive points are related to filling factors making up the hierarchy $\nu =\frac{N}{\alpha N+\beta }$. The latter matches quite well the FQHE hierarchy in consecutive subbands of LLs, according to the scheme arising from the commensurability conditions for monolayer graphene, $\nu =b\pm \frac{l}{l(2n+1)(p-1)\pm 1}$, with *n* enumerating LL, *b* depending on *n* and LL degeneracy, first ± arising from an electron-hole symmetry and second ± from the figure-of-eight shape of the trajectory. These fractions are gradually shifted toward subband edges with growing *n* and compressed to smaller periods for higher *n*, which also agrees with the details of the observation [39]. Similarly, vertical lines appearing in the picture ($\nu =\frac{const}{B}$, N=const) might be associated with a basic filling factor set $\nu =\frac{1}{p}$.

The most convincing evidence supporting the correctness of the commensurability condition is, however, the coincidence of the related predictions with the experimental observations in bilayer graphene. The pronounced feature of these observations is the occurrence of primary fractional features with even denominators also in the lowest subband of the LLL in bilayer graphene, oppositely to monolayer one [1]. The commensurability condition for bilayer graphene reproduces perfectly the observed experimentally hierarchy, including $\nu =-\frac{1}{2}$, cf. Figure 13; as is illustrated in Figure 5 and summarized in Table 2.

Note finally that the FQHE hierarchy in bilayer systems with the characteristic even denominators holds also for bilayer 2DEG conventional Hall setups and indeed the $\nu =\frac{1}{2}$ state has been discovered there [40,41], which also agrees with the commensurability predictions.

## 5. Conclusions

The condition for commensurability of cyclotron orbits building cyclotron braid subgroups with interparticle spacing in homogeneous 2D charged systems is formulated in order to verify possibility of arrangement of correlated multiparticle Hall states. Using this commensurability condition based on the braid group approach to statistics of interacting many particle systems, the hierarchy of fractional fillings for LLs in graphene has been determined. The FQHE evolutionwith growing numbers of LLs has been described. In higher LLs the new opportunities for commensurability occur leading to the different than the ordinary FQHE(multiloop) correlated states. The fractional fillings of LLs related with this new commensurability opportunity have been identified, starting from the first LL. They are referred to FQHE(single-loop) because the related correlations are described by single-looped braids. Both the monolayer and bilayer graphene have been considered and the essential difference of related hierarchy structures has been demonstrated and described. The even denominator main line of the fractional filling hierarchy in bilayer graphene is found in agreement with the experimental observations. The confirmation of the presented hierarchy for the monolayer and bilayer graphene can be found in experimental data for both, the graphene on BN substrate as well as the suspended samples including bilayer graphene available for fillings up to sixth spin-valley subband.

The success in description of hierarchy for FQHE using commensurability conditions is linked with the nonlocal and topological character of this approach. It is known that FQHE results from non-perturbative effects of the Coulomb interaction in 2D, which give rise to gapped many-body states at special filling factors. These states have long-range entanglement and cannot be explained in a simple single-particle picture. Similarly, the composite fermion theory, effective in the LLL of conventional 2DEG and in monolayer graphene, is in fact not strictly local, utilizing the auxiliary concept of flux tubes, or vortices, fixed to particles and allowing via this multiparticle effective trick to discriminate highly nonlocal effects. The commensurability approach presented in this paper bases on the topological braid group description of statistics in many body systems and falls to the same class of nonlocal multiparticle models. It elucidates artificial assumptions of composite fermion theory and allows for the generalization of the topological approach beyond the limit of the composite fermion model. In particular, the commensurability approach is effective also in higher LLs and allows the hierarchy of FQHE to be defined quite differently than that in the LLL – in agreement with experimental observations. Utilizing the energy minimization methods (exact diagonalization on finite models), the difference between FQHE states belonging to various Landau levels is referred to the different form of the effective Coulomb interaction (i.e. Coulomb interaction dressed by the form factor corresponding to a given Landau level). The energy minimization gives energy gaps for fractions related to special multiparticle correlations of FQHE. These fractions (and their hierarchy) can be, however, identified by a nonlocal effective-multiparticle topological method of commensurability braid approach, generalizing in this way the previous composite fermion approach from the LLL. The advantage of the commensurability condition is especially clearly visible in the case of bilayer graphene, where the states at fractions with even denominators cannot be explained by the simple composite fermion model, even in the LLL.
